# Development of a WebGIS-Based Analysis Tool for Human Health Protection from the Impacts of Prescribed Fire Smoke in Southeastern USA

**DOI:** 10.3390/ijerph16111981

**Published:** 2019-06-04

**Authors:** Yongtao Hu, Ha Hang Ai, Mehmet Talat Odman, Ambarish Vaidyanathan, Armistead G. Russell

**Affiliations:** 1 School of Civil and Environmental Engineering, Georgia Institute of Technology, Atlanta, GA 30332, USA; yh29@mail.gatech.edu (Y.H.); ted.russell@gatech.edu (A.G.R.); 2 School of Computing, Georgia Institute of Technology, Atlanta, GA 30332, USA; hangha1812@gmail.com; 3 National Center for Environmental Health, Centers for Disease Control and Prevention, Atlanta, GA 30333, USA; dvq3@cdc.gov

**Keywords:** air quality, exposure, forecasting, wildland fire, DDM-3D, source impact, burn acreage

## Abstract

We have developed the Southern Integrated Prescribed Fire Information System (SIPFIS) to disseminate prescribed fire information, including daily forecasts of potential air quality impacts for southeastern USA. SIPFIS is a Web-based Geographic Information Systems (WebGIS) assisted online analysis tool that provides easy access to air quality and fire-related data products, and it facilitates visual analysis of exposure to smoke from prescribed fires. We have demonstrated that the information that SIPFIS provides can help users to accomplish several fire management activities, especially those related to assessing environmental and health impacts associated with prescribed burning. SIPFIS can easily and conveniently assist tasks such as checking residential community-level smoke exposures for personal use, pre-screening for fire-related exceptional events that could lead to air quality exceedances, supporting analysis for air quality forecasts, and the evaluation of prescribed burning operations, among others. The SIPFIS database is currently expanding to include social vulnerability and human health information, and this will evolve to bring more enhanced interactive functions in the future.

## 1. Introduction

The relative contribution of biomass burning to local air quality in the USA has increased over the past decade, due to enhanced regulatory controls on anthropogenic emissions [1] and increasing wildfires, prescribed forest fires, and agricultural burns [2]. Exposure to biomass-burning smoke can be hazardous, and this poses a significant public health threat [3]. Wildfire smoke can travel long distances and become spread out; it can affect very large expanses; therefore, it can affect millions of people living downwind [4,5]. Smoke from any individual prescribed burn has a much smaller and usually local impact, although occasionally, downwind impacts can be substantial [6]. However, chronic exposure to prescribed fire smoke in places where burning is heavily practiced, such as southeastern USA, can be just as important as acute exposure to wildfire smoke. In the USA, 2.4 million acres of forests were treated by prescribed fire in 2014 [2]. The prescribed fire-treated forestry acreage increased to 6.4 million nationwide in 2017, approximately two-thirds of which are in the southeast, covering the states of Alabama, Florida, Georgia, North Carolina, South Carolina, and Tennessee. In addition, about 80 % of the 3 million acres of agricultural burning nationwide also takes place in the southeast. As a result, prescribed burning is the largest source of particulate matter with an aerodynamic diameter less than 2.5 µm (PM_2.5_) emissions in the southeast (according to NEI 2014, 20% or 210 Gg of PM_2.5_ emissions were from prescribed burns). The use of prescribed burning for forest and crop health has economical and ecosystem-related benefits, but it must be weighed and managed with respect to the potential health and welfare impacts, and the concerns over those activities leading to air quality non-attainment.

Georgia Institute of Technology has developed an advanced prescribed fire impact forecasting system—HiRes2, to provide daily forecasts of potential prescribed fire impacts in Georgia [7]. As will be described in Section 2.2, Hires2 employs well-established models for meteorological and air-quality forecasting. The forecasts of prescribed fires are based on meteorological and wildland characteristics such as winds, precipitation, fuel humidity, fuel amount information, etc. [7]. The HiRes2 forecasting products are currently used by the Georgia Department of Natural Resources (GaDNR) to provide official forecasts of air quality for public health protection, and they are also available publicly for use by other state agencies, such as the local forest service offices, for prescribed burning management.

There is an increasing demand for easy access to information on smoke exposure from wildland fire for public awareness, and for management and research. On the other hand, the recent rapid developments of Web-based Geographic Information Systems (WebGIS) and companion online visualization technologies have paved a broad avenue for building tools to meet such needs in an efficient way. For example, National Oceanic and Atmospheric Administration (NOAA) operates a website [8] that brings together satellite-based fire detection, cloud, smoke, and aerosol optical thickness data, with ground-level PM_2.5_ observations for forest and environmental management, as well as research communities, to explore the impacts of fires. The Centers for Disease Control and Prevention (CDC) is developing an online tool for identifying populations that are vulnerable to wildfire smoke hazards [3]. These efforts have created online interactive tools for convenient access to helpful fire-caused smoke-related information, but a system that combines fire and air quality observations with forecasts, focusing specifically on prescribed fires, did not exist.

This study aims to directly convey the HiRes2-produced prescribed fire impact forecasts, along with other useful related data, to various users through an online interactive tool for efficient data dissemination, and for a better analysis of air quality and human exposure, as affected by prescribed fires. WebGIS-based online tools have been developed for emissions inventory spatial analysis [9], marine pollution monitoring and forecasting [10], fire danger forecasting [11] and urban air quality monitoring data visualization [12]. Here, we present our efforts in developing a similar interactive online tool for the analysis of monitored and forecasted prescribed fire impacts in southeastern USA.

## 2. Materials and Methods 

### 2.1. SIPFIS: A WebGIS-Based Analysis Tool

**Figure 1:**
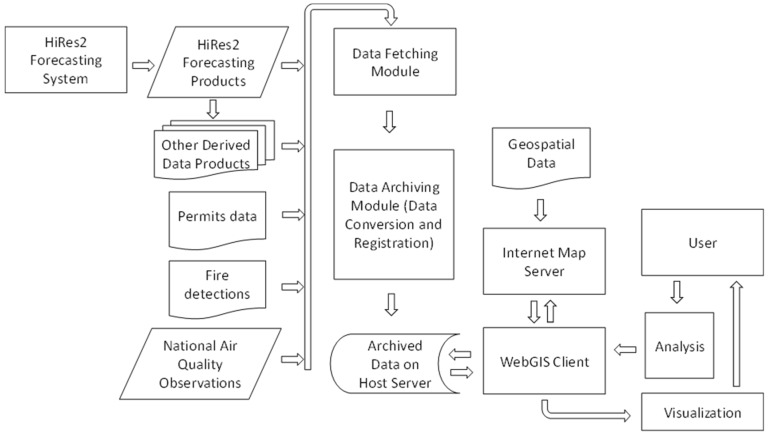
Conceptual architecture of the Southern Integrated Prescribed Fire Information System (SIPFIS) system.

The Southern Integrated Prescribed Fire Information System (SIPFIS) is a WebGIS-based online analysis tool. The current focus of the tool are the impacts of prescribed fire smoke on air quality and human health. Figure 1 shows a flowchart describing the conceptual architecture of SIPFIS. The major components of SIPFIS include the HiRes2-prescribed fire impacts-forecasting system—an in-house tool that produces air quality and prescribed burn impact data as a result of operational forecasting and other derivative products such as smoke exposure data, etc., a data-fetching component that automatically obtains outside datasets, a data-archiving component that performs data format conversion, Quality Assurance/Quality Control, and data management, and a data visualization and analysis component that directly meets the end user’s requests for interactive online analyses.

### 2.2. Prescribed Fire Impact Dataset from the HiRes2 Forecasting System

The HiRes-2 forecasting system has been in operation since 2015 for air quality forecasting in southeastern USA, over a domain centered on Georgia. The system simulates meteorology and air quality, using the Weather Research and Forecasting model (WRF, version 3.6, National Center for Atmospheric Research, Boulder, Colorado) [13], combined with the Community Multiscale Air Quality (CMAQ, version 5.0.2, U.S. Environmental Protection Agency, Research Triangle Park, NC, USA) [14] model. In addition, it provides daily forecasts of the potential contributions of prescribed fires to air quality, using the decoupled direct method (DDM) in three dimensions (DDM-3D) [15], which is a sensitivity analysis technique that is integrated within CMAQ for simultaneously computing sensitivity coefficients while air pollutant concentrations are computed.

The burn activity, i.e., the number, size, and location of burns, is forecasted using the weather forecast as input to a classification and regression tree (CART) model built from the meteorological data and burn activity of recent years. The spatial coverage of this forecast, formerly limited to Georgia, has recently been expanded to other states in southeastern USA. The burn activity data for the CART are obtained from burn permit records in Georgia and a satellite-based product [16] that provides burn locations and areas in other states. Since the satellites cannot always detect the burns in southeastern USA, due to factors such as the small size of the burns, their low heat intensity, overlaying canopy in understory burns, and frequent cloud cover [17], the burn activity forecasts in other states are not as accurate as in Georgia. Detailed information for how the CART model is built, and how prescribed fire emissions are estimated can be found in Odman, Huang et al. [7]. For each county in southeastern USA, monthly varying daily average burn areas and typical burn sizes are used to determine the number of burns on a forecasted “burn day”, and those burns are distributed randomly to the forested areas of the county. Then, fire emissions are estimated by using the fuel loads, in mass per unit area of the burn plots, obtained from Fuel Characteristic Classification System (FCCS) maps, along with fuel consumption estimates from the CONSUME model [18], based on forecasted fuel moistures and other meteorological parameters relevant to the burn, and emission factors, in terms of the mass of pollutant emitted per unit mass of fuel consumed, derived from field measurements at various locations in southeastern USA. Finally, the plume height is calculated by using an empirical model [19], based upon fire weather information such as winds, planetary boundary layer (PBL) height, fuel moisture, etc., and the emissions are distributed vertically into the CMAQ layers for air quality and burn impact forecasting.

The HiRes2 forecasting system generates burn activity, air quality with/without burns, and burn impact forecasts, on a daily basis. The air quality forecasts without burns have a 72-h lead time, while the burn impact forecasts have a 24-h lead time.

Based upon the prescribed burn air quality impact forecasting products, we also developed smoke exposure and health impact-forecasting products. One of these products weighting PM_2.5_ concentrations with the CDC’s Social Vulnerability Index (SVI) [20] is described in Appendix A. Further, we developed predictions of increased relative rates of potential emergency department visits (EDV) and hospitalization of asthma and chronic obstructive pulmonary disease (COPD) patients due to the forecasted smoke exposure. Following Vaidyanathan, et al. [3], we calculate the fractional change in health rate as follows:

(1) $$\frac{\Delta H}{H_{0}}=1-e^{-\beta \Delta C}$$

Here, ΔH is the change in the rate of the adverse health outcomes caused by ΔC, which is the forecasted prescribed burn’s impact on the PM_2.5_ concentration, $H_{0}$ is the base health rate, β is the coefficient of effect estimate obtained from the concentration–response function (C-R function). We adopted the C-R function for smoke-impacted EDV from Alman et al. [21], with β=0.008 for asthma and β=0.010 for COPD, respectively, and the C-R function for smoke-impacted hospitalization from Gan et al. [22] with β=0.008 for both asthma and COPD.

### 2.3. Other Datasets and the Data-Fetching Component

Other than the above forecasting and derivative products generated by HiRes2 and transferred to the database hosting server, the SIPFIS system also fetches the following third-party datasets:

- Air quality observations: Ozone and PM_2.5_ observations from the national air quality monitoring network [23]; a wget-based c-shell script is used to obtain the near-real-time datasets on a daily basis.
- Fire detections: The locations of satellite fire detections from the Hazard Mapping System (HMS) Fire and Smoke Product [24]; a wget-based c-shell script is used to obtain near-real-time datasets on a daily basis.
- Permit data: Authorized burn locations and areas from Florida’s open burn authorization records and Georgia’s burn permit records; we received the datasets from Florida and Georgia’s forest service agencies every year.

### 2.4. Data Archiving Components

The air quality forecasts at the monitoring locations are ingested into an open-source relational database system (MySQL) on the database server, by using a Hypertext Preprocessor (php) program. The burn activity forecasts are directly stored in text format, while the spatial forecasting products, including the air quality with/without burns and burn impact forecasts, are stored there in an open standard format (GeoJSON) that is designed for representing geographical attributes. The archived ozone and PM_2.5_ air quality forecasts are for contiguous USA, and are available for online inquiry from 1 January 2015 to present. The archived burn-related forecasting products are for southeastern USA, and they are available from 1 January 2015 to 30 April 2015, 1 January 2016 to 30 April 2016, and 1 January 2017 to present.

Similarly, the air quality observational data are ingested into the MySQL database tables by using a php program immediately after being fetched onto our database server. The archived ozone and PM_2.5_ observations are nationally available for online inquiry from 1 January 2015 to present.

The HMS detection datasets are transformed and stored in daily files in a simple text format, and they are archived on the database hosting server. The archived HMS detections are for North America, which are available online from 1 January 2015 to present.

Permit data undergo several QA/QC checks, during which missing items from each record will be filled, if possible. For example, the latitude and longitude of the burns, if not provided, are derived from the provided addresses by utilizing Google Earth services. Finally, the checked records are ingested into MySQL database tables, while inaccurate or incomplete records are discarded. Currently, archived permits data are for Florida and Georgia, available from 1 January 2015 to 31 December 2016. The datasets current archived in SIPFIS are summarized in Table 1.

### 2.5. Data Visualization and Analysis Components

To facilitate the visual analysis, we developed three methods of displaying the data per user’s request, through the web server. The first two methods present the data either as polygons (contours) or as point markers on the map. The background map is provided through OpenStreetMap [25], which supports spatial navigation, and can zoom in for very detailed local geographical information. The spatial distributions of the air quality index (AQI) grades or the burn impacts are presented through the Leaflet function for polygons in the GeoJSON format [26]. Points of air quality observations, HMS detections, burn locations and permits data are presented on the map as various markers, through a Leaflet pointToLayer function. In the third method, site-specific observations and forecasts are presented, using time-series plotting functions from the D3.js library [27]. All the above displaying components are realized by using Javascript functions, and they support interactive capability to display data according to the selected date, pollutant (i.e., ozone or PM_2.5_), and/or data type. A user’s manual describing how various visual analyses are conducted with SIPFIS, using its interactive functions, can be found in Appendix A.

## 3. Results

**Figure A1:**
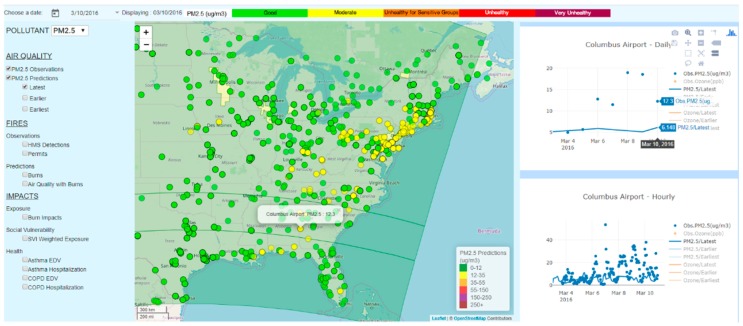
The SIPFIS webpage displays, as an example, PM_2.5_ observations for 10 March 2016, overlaid with the latest PM_2.5_ forecasts, as well as the time series for the monitoring site at Columbus.

**Figure A4:**
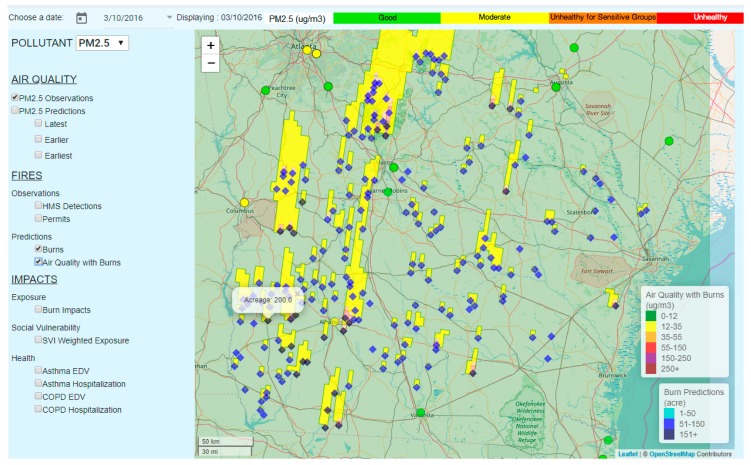
SIPFIS webpage displays forecast burns, overlaid with PM_2.5_ forecast levels including the impact of burns.

**Figure A5:**
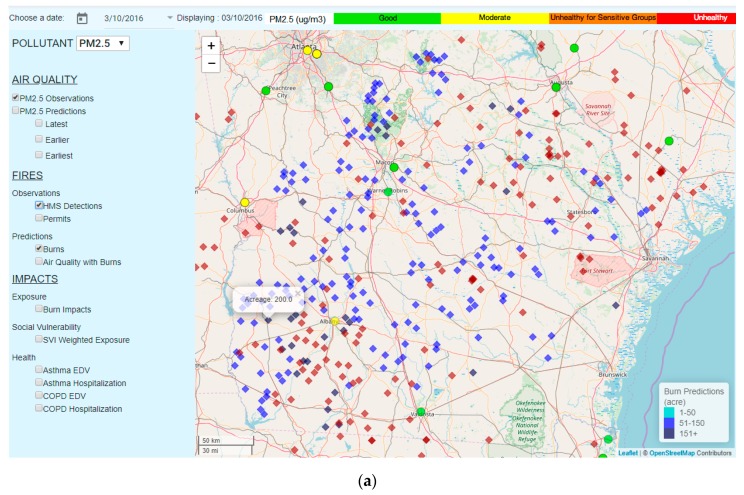

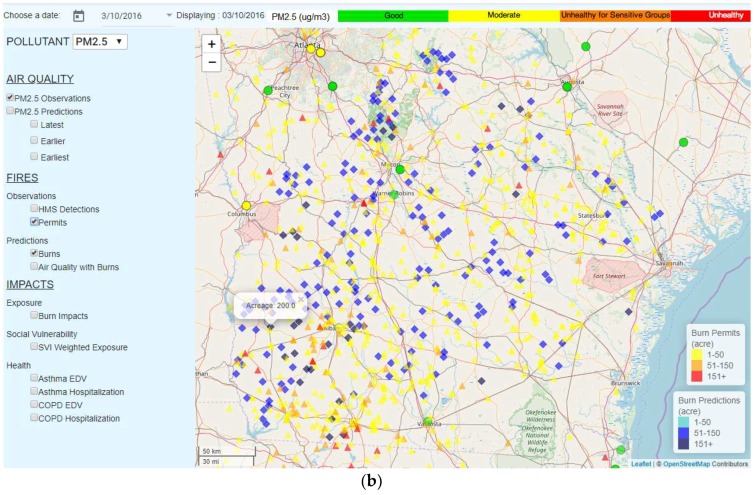
The SIPFIS webpage displays (**a**) HMS detections (burgundy diamonds) and (**b**) permits (yellow, orange, and red triangles), with acreage information.

Several types of analyses can be performed with the current interfaces of SIPFIS. These include fire analyses comparing the locations of permitted burns to locations of satellite-detected fires, the areas of the permitted burns to the satellite-derived burned areas, and forecasts of the burn activity to permits and satellite fire detections. As an example, the comparison among HMS detection, permits, and forecast burns on 10 March 2016, is shown in Appendix A (Figure A5). SIPFIS can be used to access various forecasting products for personal use, such as residential community level or mobility-based air quality forecasts. As an example, starting with the forecast displays in Figure A1 and Figure A4, a user can further zoom into the background map to residential community levels in Georgia, and by changing the dates, the AQI grades around their neighborhood for the next three days can be obtained. SIPFIS can also be used as a screening tool for fire-related exceptional events that led to exceedances and violations of air quality standards, or a supporting analysis tool to assist and evaluate air quality forecasts. Forest- and air quality managers, as well as public health practitioners in health departments, can also use SPIFIS to evaluate prescribed burning operations that aim for minimal human exposure to fire smoke. Here, the applications for four such analyses are illustrated in detail by the examples below.

### 3.1. Assisting the Air Quality Forecast and Its Evaluation

**Figure 2:**
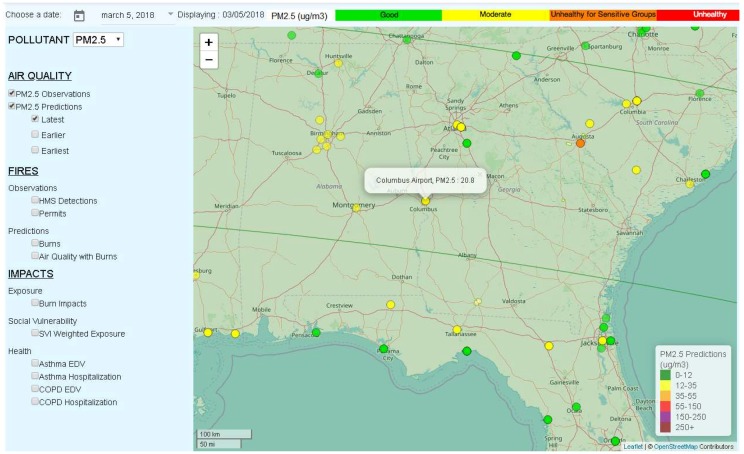
PM_2.5_ observations and HiRes2 forecasts at the Columbus Airport site, and in the region surrounding Columbus, Georgia, on 5 March 2018.

As mentioned above, to protect public health, GaDNR issues daily PM_2.5_ forecasts for Atlanta and Columbus, GA, and during the ozone season (April–October), ozone forecasts for Atlanta and Macon, GA. The official forecasts for the next day are released at around 13:45 local time, and they represent the consensuses reached by a team of experts. The HiRes2 air quality forecasting products are among the various tools that are available to the experts, for supporting their decision. On 5 March 2018, the official GaDNR forecasts for PM_2.5_ in Atlanta and Columbus, GA were 11 and 8 µg/m^3^, respectively, while the recorded 24-hr average PM_2.5_ concentrations were 20.0 and 20.8 µg/m^3^ (the AQI color was yellow for moderate, meaning that there may be health concerns for people who are sensitive to air pollution), as seen in Figure 2 (See Appendix A for how to interpret the observations and predictions that are shown on the map). The HiRes2 forecasts were 4.9 µg/m^3^ for Atlanta and 6.4 µg/m^3^ for Columbus (the AQI color was green for good, meaning that the air pollution poses no risk).

**Figure 3:**
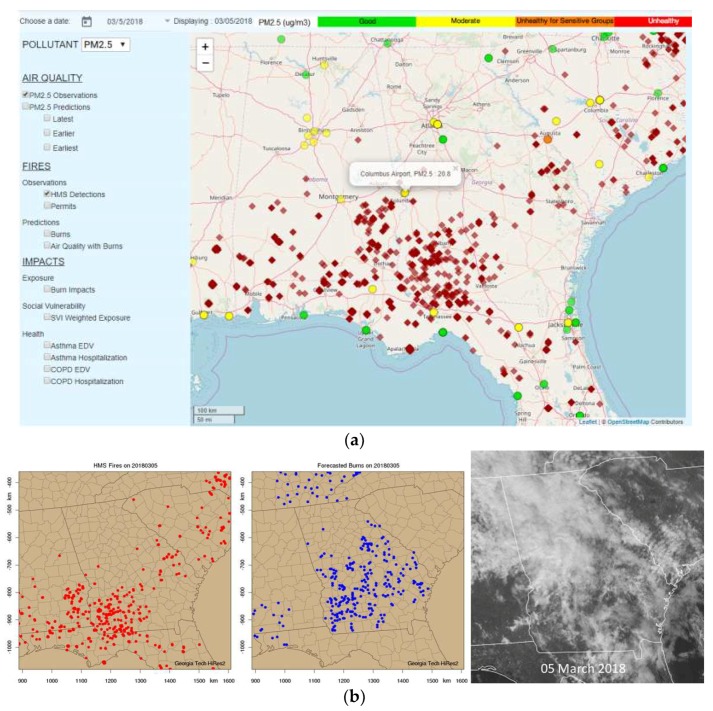
(**a**) PM_2.5_ observations and HMS fire detections in the region surrounding Columbus on 5 March 2018, and (**b**) HMS detections compared with prescribed burns that forecasted by HiRes2, along with the cloud image at 1 p.m. Eastern Standard Time.

On the same day, HMS detected numerous fires in the region (Figure 3a). There may have been other fires that were undetected because of the cloud cover. For example, in the southern portion of Tennessee, HiRes2 forecasted many burns, while satellites detected none in this area, due to the heavy cloud coverage (Figure 3b). Around Columbus, GA, which was also covered by clouds, the HiRes2 burn forecast predicted many more prescribed fires than what the satellites detected. On the other hand, the HiRes2 burn forecast outside of GA missed the burns in SC, southeastern AL, and the FL panhandle. Both the HMS fire and smoke analyses, and our burn impact forecast, suggested a heavy smoke presence near Columbus under light southerly winds.

**Figure 4:**
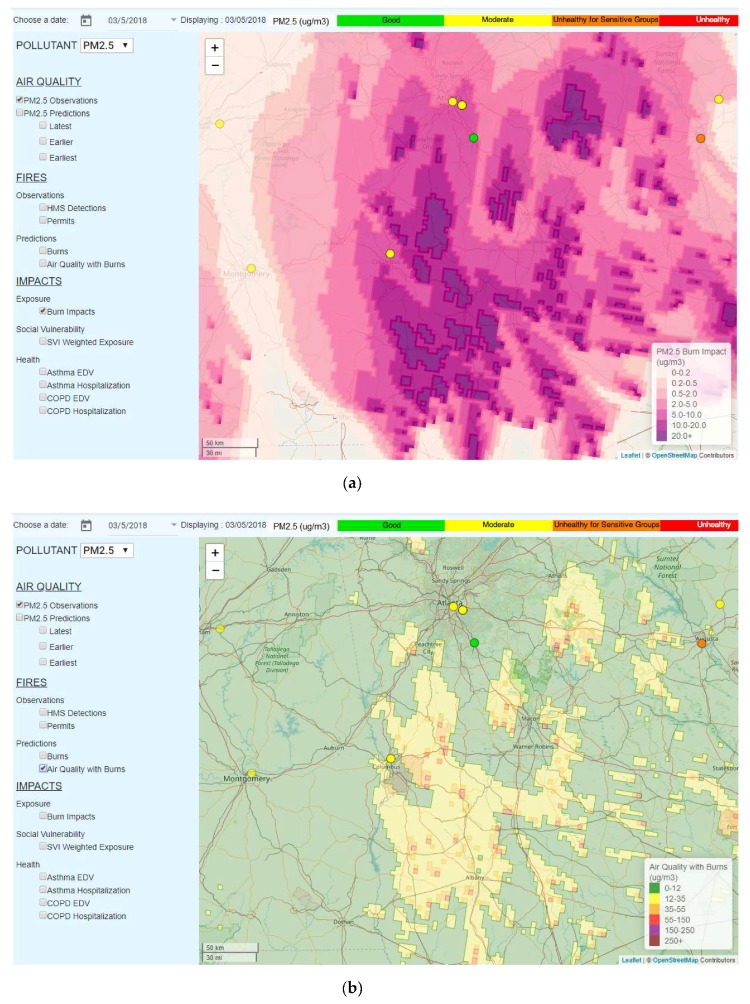
(**a**) The burn impact forecast on 5 March 2018, and (**b**) the air quality forecast after including the impact of burns.

The burn impact forecast predicted a prescribed fire contribution of 5–10 µg/m^3^ to the 24-h PM_2.5_ concentrations, both at Atlanta and Columbus, GA (Figure 4a). This was sufficient to turn the forecast for Columbus from green to yellow (see Figure 4b). Atlanta’s forecast with burns was still in the green. Assistance from the burn impact forecast would have allowed for a correct forecast of the air quality at Columbus on 5 March 2018.

### 3.2. Screening Fire-Related Exceptional Events that could Lead to Exceedances

**Figure 5:**
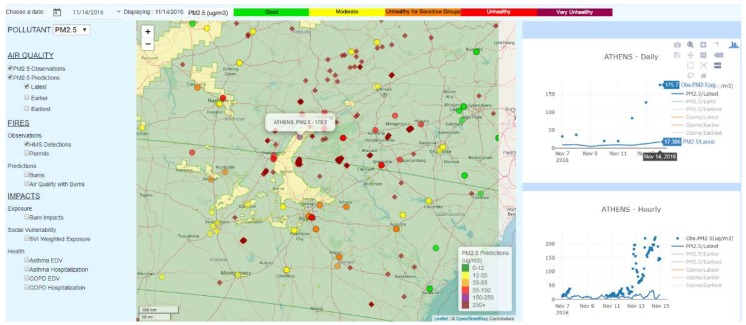
A potential fire-related exceptional event that led to PM_2.5_ exceedance in Athens, TN, on 14 November 2016.

By using SIPFIS, one can quickly conduct pre-screening for fire-related exceptional events that lead to exceedances. Here, we used the 14 November 2016 PM_2.5_ exceedance in Athens, TN, as an example. The observed PM_2.5_ concentration at Athens, TN was 175.7 µg/m^3^; however, the forecast without fire emissions only gave 17.5 µg/m^3^ (Figure 5). By checking the HMS detections, one can quickly determine that this was potentially a fire-related exceptional event for Athens, TN, on that day, and initiate further detailed investigations. HMS fire detections were concentrated heavily to the south of the monitoring site, with several other fires scattered in the surrounding areas. When there are such extensive fire spots in the region, some of them might be large wildfires. According to the U.S. Federal Rule, “Treatment of Data Influenced by Exceptional Events” (EPA 40 CFR Parts 50 and 51), wildfires qualify as exceptional events, and under certain conditions, prescribed fires could qualify as exceptional events as well. Those conditions include the use of smoke management programs (SMP), and the application of basic smoke management practices (BSMP) for prescribed fires by the states.

**Figure 6:**
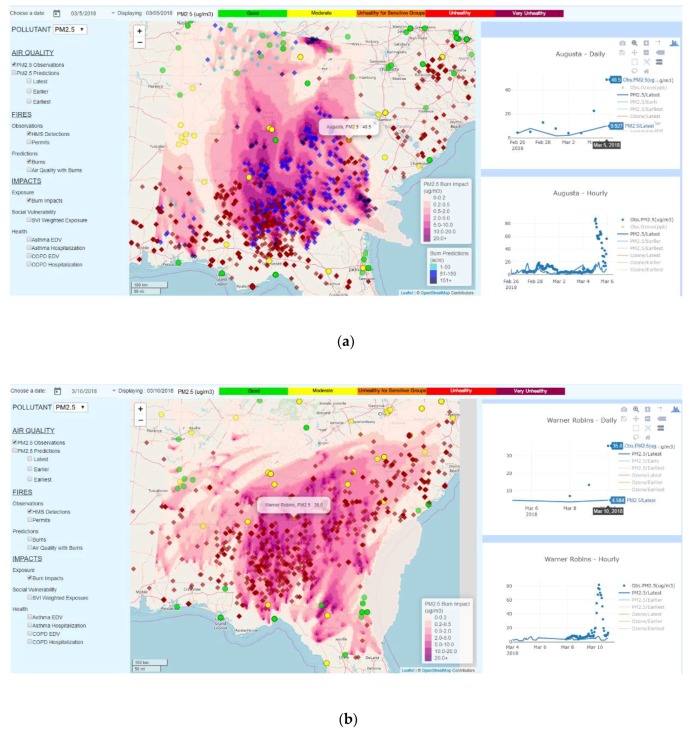
Potential fire-related exceptional events that led to PM_2.5_ exceedances (**a**) in Augusta, GA on 5 March 2018 and (**b**) in Macon, GA on 10 March 2018.

Similar potential fire-related PM_2.5_ exceedances in Augusta (48.5 µg/m^3^) on 5 March 2018 (Figure 6a), and in Macon (35.8 µg/m^3^) on 10 March 2018 (Figure 6b). The HiRes2 forecasts without fire emissions were only 9.9 µg/m^3^ and 4.6 µg/m^3^, respectively. For both events, HMS detections showed extensive fire spots in the region surrounding the monitoring site, implying potential fire impacts. For these two cases, we had additional prescribed burn impact forecasts that provided further supporting evidence. On 5 March, the forecasted fire impacts headed slightly off the direction towards Augusta, but they still made a 2 µg/m^3^ contribution. On 10 March, the forecasted fire impacts hit the monitoring site in Macon directly, resulting in an additional 5–10 µg/m^3^ PM_2.5_ concentration, strongly supporting the exceedance was fire-related, though the magnitudes of the impacts are underestimated. Note that the forecast fire locations, numbers, and sizes should not be expected to exactly match the actual fires, due to the random placement of predicted burns, and the use of typical fire sizes and daily average burn areas in the counties.

### 3.3. Assisting in the Evaluation of Prescribed Burning Operations for Minimal Smoke Exposure

**Figure 7:**
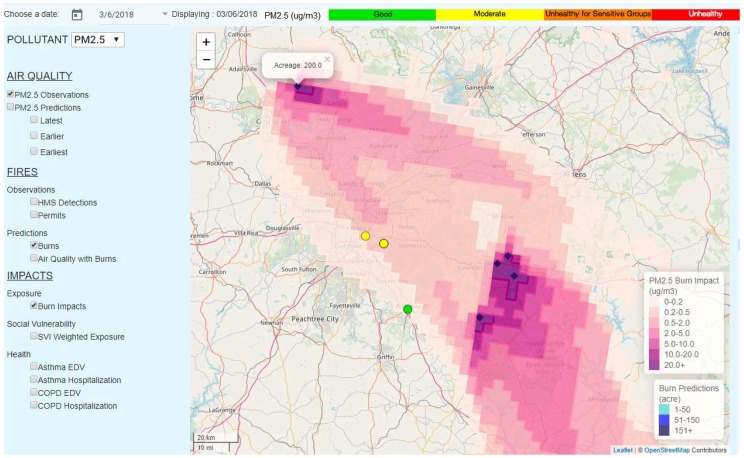
Predicted smoke impacts on populated areas on 6 March 2018.

For 6 March 2018, HiRes2 predicted several prescribed burns not too far from the Atlanta metro area, one of which was near the city of Adairsville, GA, to the Northwest of Atlanta, burning 200 acres. According to the burn impact forecast, the smoke from those burns would travel directly towards populated areas in Atlanta metro area (Figure 7). For the purpose of minimal smoke exposure, a forest manager user of SIPFIS may use this evidence to restrict or reject burning permit applications from that particular area.

### 3.4. Quantifying the Health Impacts of Exposure to Smoke from Prescribed Burns

**Figure 8:**
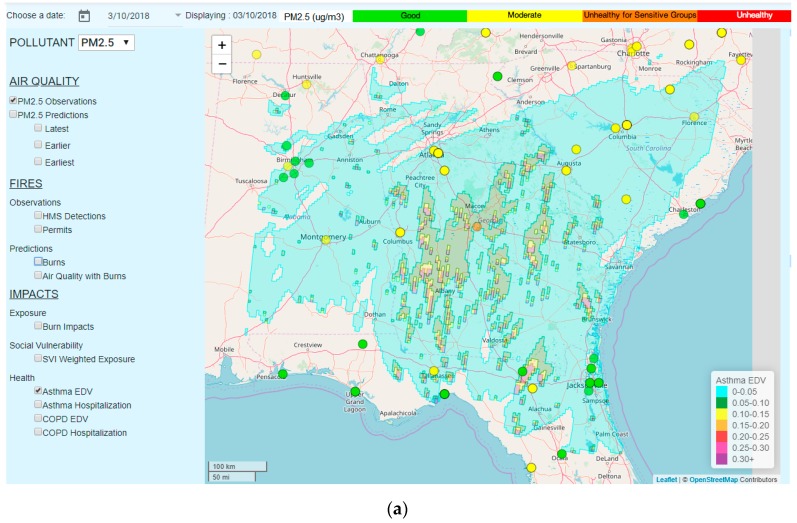

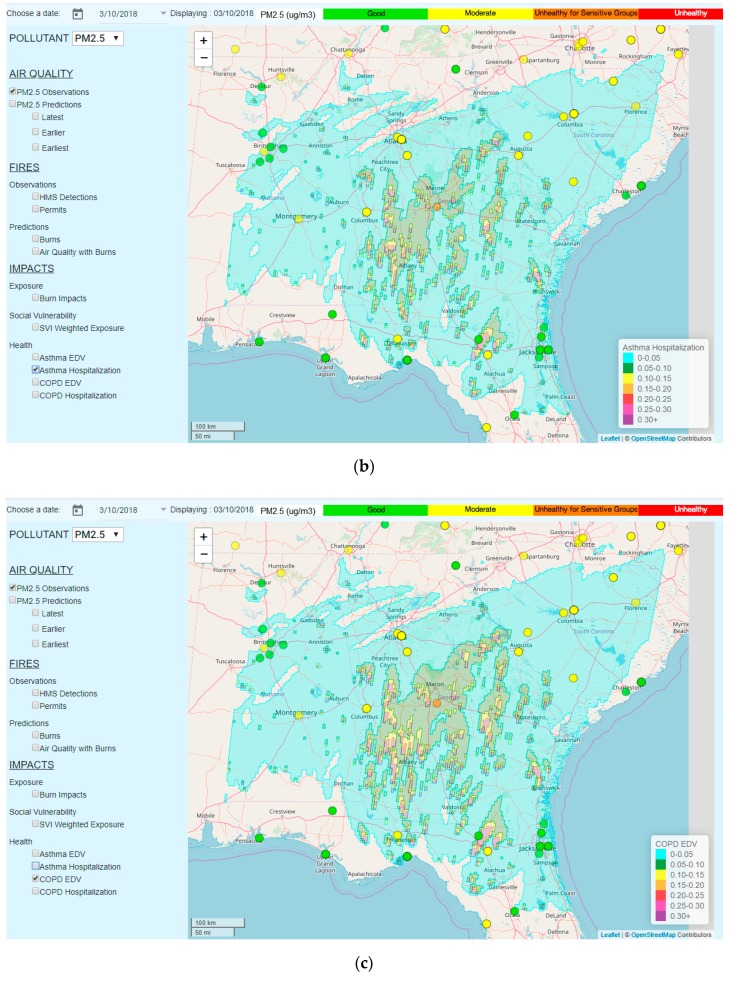

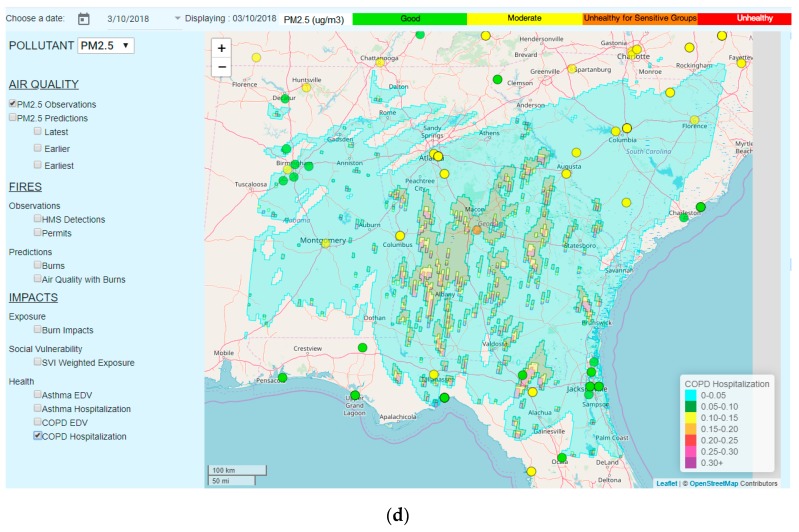
Spatial distributions of increased relative rates $\left(\frac{\Delta H}{H_{0}}\right)$ for (**a**) the emergency department visit (EDV) and (**b**) hospitalization of asthma patients, and (**c**) the EDV and (**d**) hospitalization of chronic obstructive pulmonary disease (COPD) patients due to forecasted smoke exposure on 10 March 2018.

For 10 March 2018, HiRes2 predicted extensive prescribed fires in Southern Georgia which could have increased exposures to PM_2.5_ by a large margin, especially at locations impacted by smoke plumes, and which consequently could have caused a significant increase in the number of EDV and hospitalizations of both asthma and COPD patients (Figure 8). The predicted increased rates of EDV and hospitalization were as large as ~40% at the locations shown as purple spots on the map. This information can be used by asthma and COPD patients living near the influenced areas, to limit outdoor activities on that particular day, and also by local health care professionals, to prepare in advance for increased EDV and hospitalization frequencies by asthma and COPD patients.

## 4. Discussion

Unlike other similar online tools for access to fire and smoke-related information such as the enhanced Infusing satellite Data into Environmental Applications (e-IDEA) [8], or the European Forest Fire Information System (EFFIS) [11], SIPFIS focuses specifically on prescribed fires, and it combines satellite-based fire and air quality observations with ground-based information, and provides analysis tools for direct comparisons of past forecasts with observations. It expands prescribed fire impact information beyond just air quality impacts, by providing estimates of human exposure and health effects. As such, SIPFIS can be used by forestry, air quality, and public health communities for the cohesive management of prescribed fires and their various impacts in southeastern U.S.

The current functions of SIPFIS that are available for public use [28] are being augmented. In the future, we will develop functions that will enable users to save their analysis results, including tables and plots as reports, and that can probe data values from polygons at specified spots on the map etc. We will also expand the observational database to include more satellite retrievals, such as aerosol optical depth (AOD), and CO and NO_2_ column data, etc. We will further introduce data-fused gridded concentrations products that would combine strengths from both the model-simulated concentrations and surface and space observations, to produce best-guess and full spatial coverage of surface pollutant concentrations, especially in areas with sparse observations. The system will expand to include additional states in eastern USA.

## 5. Conclusions

We have expanded the HiRes2-prescribed fire impact forecasting system, which was previously developed to provide daily forecasts of potential prescribed fire impacts on air quality in Georgia, to better serve a larger region of southeastern USA, covering parts of Alabama, Florida, North Carolina, South Carolina, and Tennessee. We have also extended it to conduct forecasts of other prescribed fire impacts, such as smoke exposure and health burdens. We have also built an SIPFIS WebGIS-based online analysis tool to bring the HiRes2-produced and prescribed fire-impact forecasts, along with other useful fire-related data products, directly to users, for more efficient access to data, and for quick analyses on the levels of smoke exposure caused by prescribed fires in the Southeast.

We have demonstrated that the current interactive functions of SIPFIS can help users to finish many prescribed fire-related data analysis tasks quickly and conveniently. These tasks include, but are not limited to: comparing burn permit records for satellite-detected fires and satellite-derived burned areas, as well as forecasts of burn activity, personal use for checking residential community-level smoke exposures, pre-screening for fire-related exceptional events that lead to exceedances, and supporting analyses for air quality forecasts, and the assistance and evaluation of prescribed burning operations, with minimal human exposure to fire smoke.

## Figures and Tables

**Table 1:** Archived datasets and their available time ranges.

Datasets	Source	Available Time Range
Air quality forecasts at sites	HiRes2	1 January 2015 to present
Burn activity forecasts	HiRes2	1 January 2015 to 30 April 2015; 1 January 2016 to 30 April 2016; 1 January 2017 to present
Spatial air quality forecasts without burns	HiRes2	1 January 2015 to 30 April 2015; 1 January 2016 to 30 April 2016; 1 January 2017 to present
Spatial air quality forecasts with burns	HiRes2	1 January 2015 to 30 April 2015; 1 January 2016 to 30 April 2016; 1 January 2017 to present
Spatial burn impact forecasts	HiRes2	1 January 2015 to 30 April 2015; 1 January 2016 to 30 April 2016; 1 January 2017 to present
Air quality observations	AirNow	1 January 2015 to present
Hazard Mapping System (HMS ) detections	NOAA	1 January 2015 to present
Permits data	Florida and Georgia Forest Service Agencies	1 January 2015 to 31 December 2016
Spatial Social Vulnerability Index (SVI)—weighted smoke exposure forecasts	HiRes2	1 January 2019 to present
Spatial forecasts of increased relative health rates due to smoke exposure	HiRes2	1 January 2019 to present

**Figure A2:**
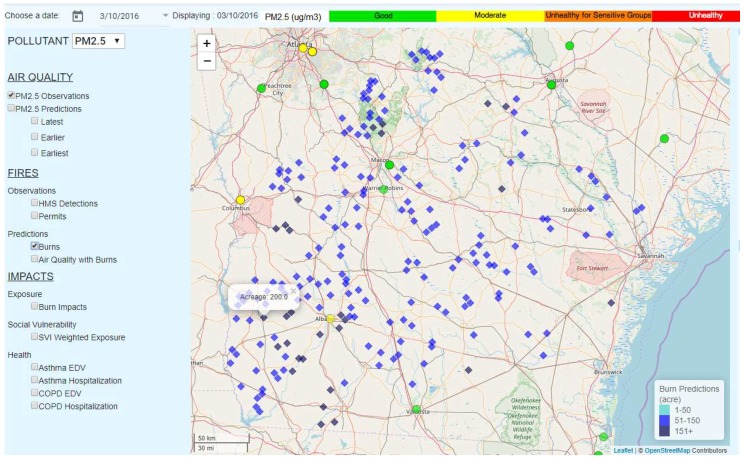
The SIPFIS webpage displays Forecast Burns as blue diamonds (in three different shades).

**Figure A3:**
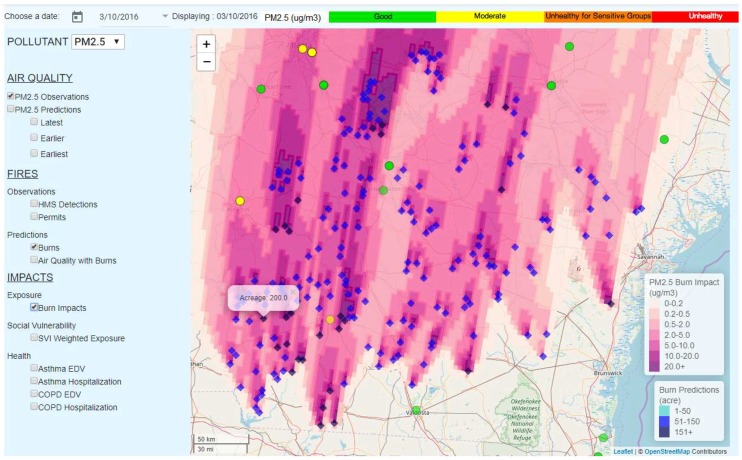
The SIPFIS webpage displays forecasted burns as blue diamonds (in three different shades), overlaid with burn impact forecasts (i.e., increased PM_2.5_ concentrations due to burn impacts).

**Figure A6:**
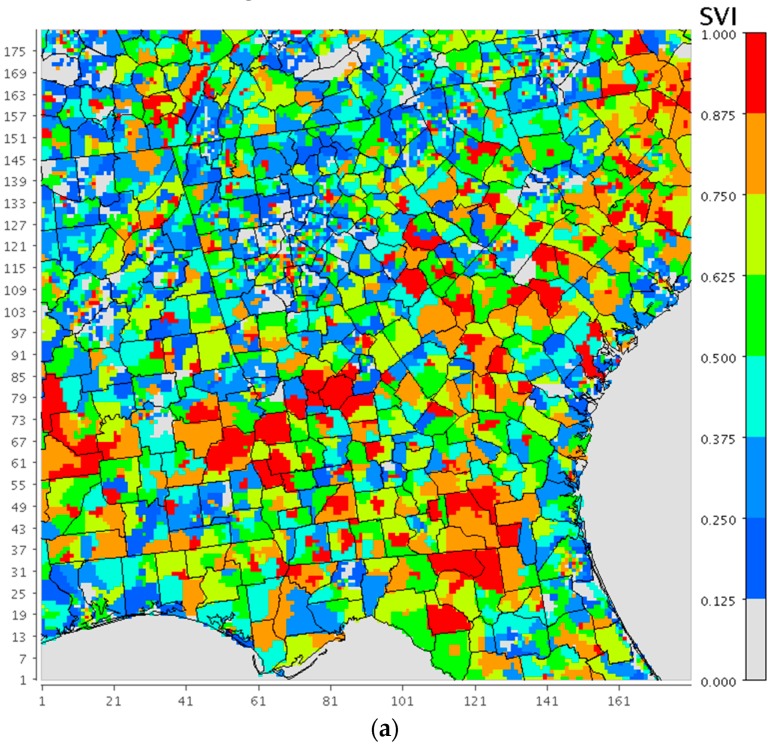

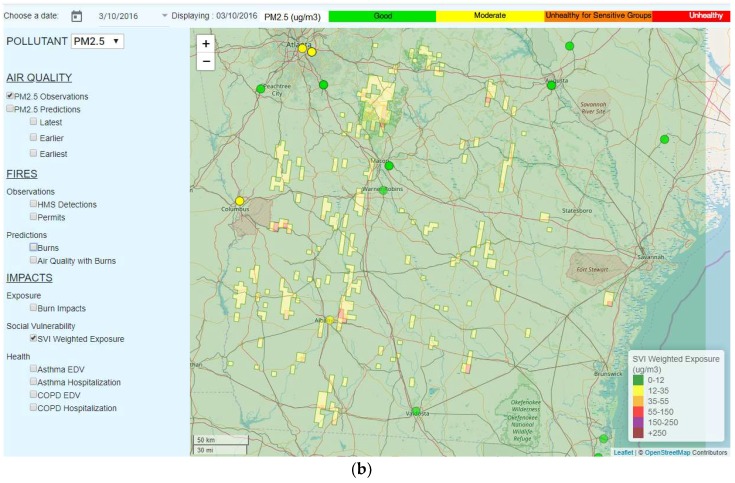
(**a**) Spatial distribution of the SVI index over southeastern U.S., and (**b**) the SVI-weighted exposures to smoke causing increases in PM_2.5_ levels on 10 March 2016.

**Figure A7:**
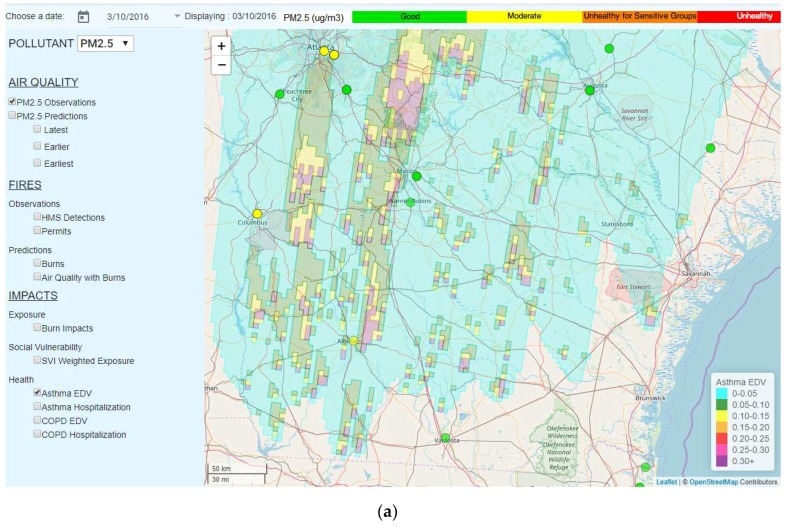

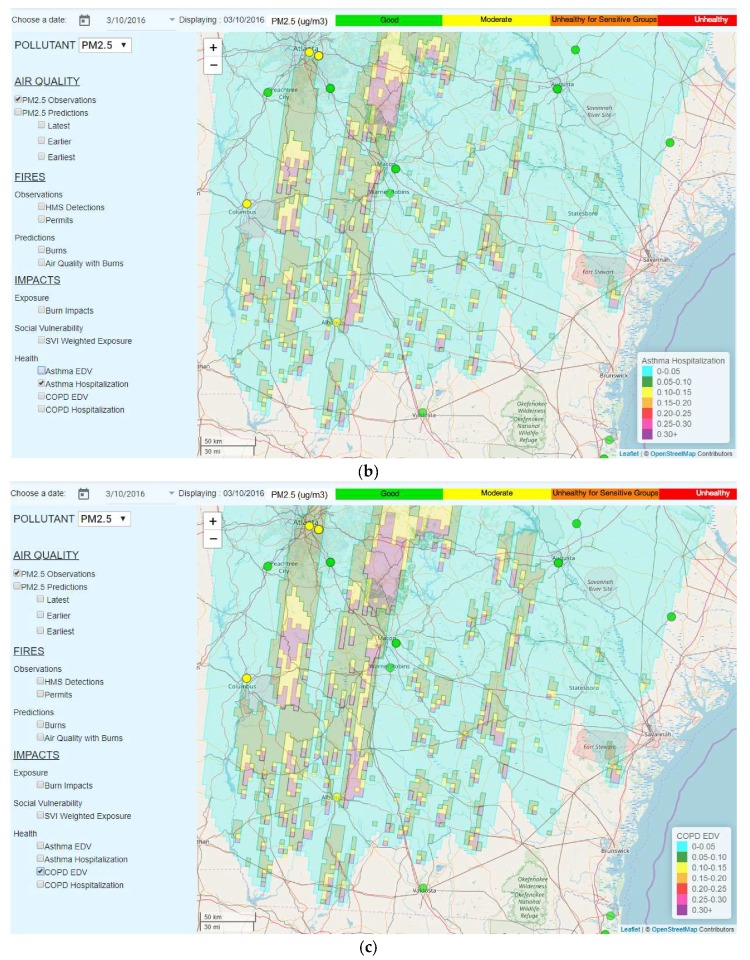

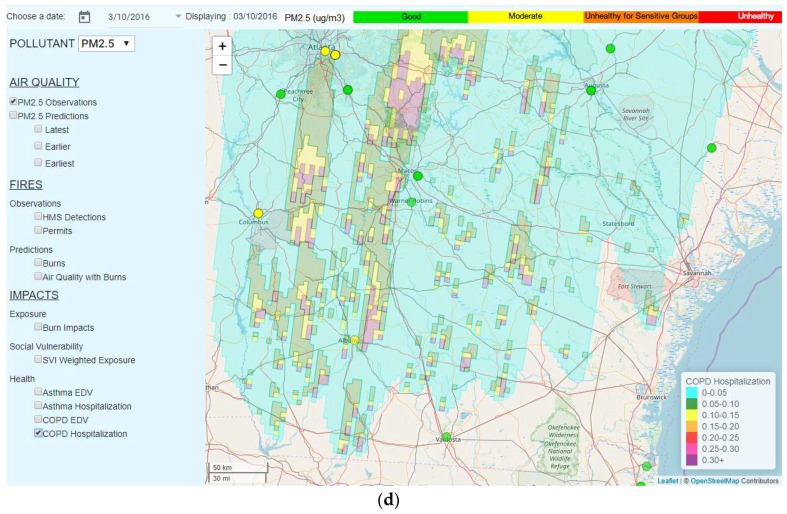
Spatial distributions of increased relative rates $\left(\frac{\Delta H}{H_{0}}\right)$ for (**a**) EDV and (**b**) the hospitalization of asthma patients, and for (**c**) EDV and (**d**) the hospitalization of *COPD* patients, due to the forecasted smoke exposure on 10 March 2016.

## Appendix A. The SIPFIS User’s Manual

The WebGIS-based SIPFIS provides access to the archived data, and facilitates visual analyses. The current URL is https://sipc.ce.gatech.edu/SIPFIS/map/ [28]. We recommend using Microsoft Edge or Google Chrome as the browser. The archived data can be accessed and analyzed interactively through several user-friendly functions, as described below.

The website opens with a display of yesterday’s PM_2.5_ observations in a region centered on Atlanta, with Confederate Avenue as the selected site. The following actions can be performed:

1. Enter a different date or select a day from the calendar, starting from 1 January 2015.
2. Select a different pollutant. The pollutant choices are PM_2.5_ (default) and ozone.
3. Zoom in or out on the map, or move to a different region.
4. Select a different site on the map by clicking on the circular marker (Figure A1). The names of the sites appear when hovering over their markers on the map, along with the most relevant value of the selected pollutant for that day (the daily average PM_2.5_ or the daily maximum 8-h average ozone concentration). The circular marker is colored according to the color code for the air quality index that is displayed next to the pollutant name at the top of the map.
5. Click on “Click to Open Time Series” on the right-hand-side of the web page to display time series plots for a selected site. There are two time series: (1) daily and (2) hourly, both for a week-long period preceding the selected date. The observed and forecast pollutant concentrations are shown for comparison. Clicking on the markers or lines in the legend makes those observations or forecasts appear or disappear. Hovering over the time series plots displays the concentration values for a particular day. To zoom in to a particular section of the graph, left-click and drag the mouse over that section; double click on the graph to zoom out.
6. Check the box for “PM_2.5_ Forecasts” on the left-hand-side menu to overlay the latest PM_2.5_ forecast field on the map (Figure A1). Note that there are three different forecasts: (1) Latest, (2) Earlier, and (3) Earliest. We forecast the air quality every day for the next three days. The latest forecast is the one from the previous day’s forecasting cycle. The next forecast is from the forecast issued two days before, and the earliest is three days prior. Note that this particular forecast does not include the potential contributions of prescribed burns.
7. Check the box for “Burns” under “Predictions” on the left-hand menu to display forecasted prescribed burns. The blue diamonds mark the locations of forecasted burns. Hovering over the burns displays their acreage. For example, there was a forecasted 200 acre burn to the south of Columbus on 10 March 2016 (Figure A2).
8. Check the “Burn Impacts” box to see the forecasted prescribed burn impacts on PM_2.5_ (or ozone) levels (Figure A3).
9. Check the box “Air Quality with Burns” to overlay the map of the predicted air quality with the prescribed burn emissions added (Figure A4).
10. To investigate whether there were actual burns in the area, the user can check the “HMS Detections” and “Permits” boxes (Figure A5). HMS data on the website consists of satellite-detected fire locations only (burgundy diamonds); there is no other information associated with these fires. The permits (yellow, orange and red triangles) provide acreage information. This provides additional evidence for the accuracy of the burn and burn impact forecasts.
11. Check the box for “SVI-weighted exposure” under “Impacts” on “Social Vulnerability”, on the left-hand-side menu, to show spatial distribution of the SVI-weighted exposures to smoke causing increased PM_2.5_ levels (Figure A6).
12. Check the box for “Asthma EDV”, “Asthma Hospitalization”, “COPD EDV”, or “COPD Hospitalization” under “Impacts” on “Health” on the left-side menu, to show the spatial distributions of the increased relative rates, respectively, for EDV, and the hospitalization of asthma or EDV, and of COPD patients due to the forecasted smoke exposure (Figure A7).

By combining the functions provided online, users can perform different analyses freely for their own purposes.
